# Magnitude of parasitic infections and associated factors among pregnant women at health facilities in Hawassa, Southern Ethiopia

**DOI:** 10.12688/f1000research.27584.1

**Published:** 2021-02-17

**Authors:** Demelash Wachamo, Fisseha Bonja, Bamlaku Tadege, Siraj Hussen

**Affiliations:** 1 Department of Public Health, Hawassa College of Health Sciences, Hawassa, Sidama National Regional State, 84, Ethiopia; 2 Medical Laboratory, College of Medicine and Health Sciences, Hawassa University, Hawassa, Sidama National Regional State, 1015, Ethiopia

**Keywords:** Intestinal Parasitic Infection; Pregnant Women, Hawassa, Southern Ethiopia

## Abstract

**Background:** Intestinal parasitic infections (IPIs) are common problems during pregnancy, with adverse outcomes including low birth weight and prenatal mortality. The burden of parasitic infections and its impacts are high among pregnant women in developing countries like Ethiopia. Therefore, this study aimed to assess the burden and associated factors of parasitic infections.

**Methods:** A facility-based cross-sectional study was conducted among 365 randomly selected women attending antenatal clinic at five selected health facilities. Data was collected by a pre-tested questionnaire and stool specimens were collected in clean plastic containers. A combination of direct microscopy and the formol-ether concentration technique was used as soon as the specimen collected. Data entry and analysed for descriptive and logistic regression models by SPSS v.23. The result declared as statistically significant at p < 0.05.

**Results:** The overall prevalence of IPI was 161 (45.9%). The most frequently identified parasites were *Ascaris lumbricoides* (27.9%), *Schistosoma* species (13.7%), *Trichuris trichiura* (5.1%), Hookworm (4.8%), and *Taenia* species. (1.4%). The IPIs were associated with women having no formal education [AOR=2.19, 95% CI: 1.05-4.57] or elementary school education [AOR=1.90, 95% CI: 1.11-3.27], as compared with high school educated and above. Monthly income of less than 1920 Ethiopian birr [AOR=2.06, 95% CI: 1.28-3.31], sharing a latrine with neighbours [AOR=1.83, 95% CI: 1.14-2.93], using lake water for washing clothes [AOR=2.24, 95% CI: 1.34-3.74], habit of eating raw vegetables [AOR=2.26, 95% CI: 1.30-3.92] were associated with IPI as compared to their counterparts.

**Conclusions:** Nearly half of the pregnant women were infected with IPs. The health facilities and clinicians need to focus on prevention of IPIs by early diagnosis, treating lake water before use, promote proper latrine utilization and provision of pertinent health education as part of ante-natal care service. It is important to minimize the impact of IPIs on pregnant women and their child.

## Introduction

Intestinal parasitic infections, mainly soil-transmitted helminths, and water-related parasitic diseases are is the common problem during pregnancy in low- and middle-income countries (LMICs) ^1^. More than 50% of pregnant women in LMICs suffer from anaemia, and low birth weight and prenatal mortality ^2^. *Ascaris lumbricoides*, *Trichuris trichiura*, and hookworms are the most common causes of intestinal parasitic infections (IPIs), which affect more than 2 billion people worldwide ^3^. The prevalence and impacts of these parasites are high among pregnant women in developing countries like Ethiopia ^4, 5^. IPIs were the second most predominant cause of outpatient morbidity in the LMICs, particularly in sub-Saharan Africa including Ethiopia ^6, 7^. 

Those in developing countries are more likely to suffer from IPIs due to a lack of resources, poverty, low literacy rates, lack of safe drinking water, poor hygiene, malnutrition and hot and humid tropical climate ^8^. Many pregnant women in developing countries are more vulnerable to IPIs due to poverty. In addition to this, they can’t afford a safe water supply, shoes, nutrition or sanitation practices ^7, 9^. 

However, the Transformation Plan, the Health Sector Transformation Plan, the Neglected Tropical Diseases (NTDs) Master Plan, the National Hygiene and Environmental Health Strategy, and the targets of the One WASH National Programme working to reduce NTDs or parasitic disease ^10^. Even though nationally representative and comprehensive data regarding the magnitude of prevalence of IPIs among pregnant women lack in Ethiopia, There is high burden of parasitic infection in Ethiopia; some regional studies have shown the prevalence ranging from 27% to 70.6% in Ethiopia ^4, 11^. The main identified IPIs are *Ascaris lumbricoides*, Hookworms, *Trichuris trichiura* and *Schistosoma mansoni* ^12^. Therefore, it is crucial to identify the magnitude of IPIs and their determinant factors to reduce the burden and impacts of these parasites on pregnant women and their children. This is important for researchers, clinicians, and health planners.

This study sought to assess the magnitude of intestinal parasitosis in the study area, which of the identified species was the most dominant, and which of the risk factors are pregnant women mainly exposed to for intestinal parasitosis in the study area.

## Methods

### Study setting and study population

A facility-based cross-sectional study was conducted among pregnant women attending antenatal care (ANC) clinics at five selected health centres in Hawassa, in Southern Ethiopia from September 01, 2019, up to Jan 30, 2020, which is one of the most densely populated areas in Ethiopia. The source of the population was all pregnant women who visited five selected health centres. All pregnant women attending ANC clinics within the study period were considered as the study population. Randomly selected pregnant women who attend ANC clinics at five randomly selected functional selected health centres, who gave informed consent and were resident in the study area were included in the study. While, those who had been undergoing anti-helminth treatment for the last one month, and those who were seriously ill during the study period were excluded from the study.

### Sample size and sampling technique

The required sample size (n) was 365, calculated by using single population proportion formula with the assumptions: by taking previously conducted prevalence of IPIs (p) (31.5%) among pregnant women attending ANC at Felege Hiwot Hospital, northwest Ethiopia ^9^, with 95% CI (1.96), 5% margin of error (d), and addition of 10% contingency:

$$n={\left(Z_{1-\alpha /2}\right)}^{2}\frac{P[1-P]}{d^{2}}\;n=\frac{{\left(1.96\right)}^{2}(0.315)(0.685)}{{\left(0.05\right)}^{2}}=331.57$$

The final sample size was adjusted as follows:

Sample size = n (sample size) + (10% non-respondent)

Thus, final sample size (n) was calculated as n= 331.57 + 33.2 = 364.77 ≈ 365.

The random sampling technique was used to select five public health facilities among 12 functional health facilities with ANC service. The desired number of clients for each health facility was selected based on proportional sampling. The study participants were selected from each facility were by the random arrival of the pregnant women to an ANC clinic.

### Study variables

The dependent variable of the study was the magnitude of IPIs, it was determined by a combination of direct microscopy and the formol-ether concentration technique (diagnosed at least one or more parasitic infections). Independent variables measured in the study were socio-demographic and economic variables (such as; age, sex, marital status, educational level, occupation, income), environmental and behavioural characteristics (housing condition, ownership and type, waste refuse, drinking water availability, living with domestic animal and pets, and latrine utilization, hand washing practice, and utilization of the lake water).

### Data collection tools, and procedures

Data was collected by using a pre-tested questionnaire to obtain socio-demographic information and pregnancy-related factors (available as *Extended data*) ^13^. The English questionnaires were translated to local language by another expert using properly designed and pretested questionnaire. The questionnaires were pre-tested and validated two weeks before in the study time on 5% of ANC attendants at a health center which was outside of the study area. Finally, some modifications on sequence or arrangement of multiple answer questionnaire and missed information were edited. The data were collected by five trained nurses educated to diploma level, five Laboratory technicians and supervised by two nurses educated to BSc level. Data was collected after explaining the objective of the study, rights and responsibilities of giving information and ascertain their confidentiality to minimize information bias. Midwifery nurses who can speak the local language were trained for data collection procedures to attain standardization and maximize interviewer reliability. In addition to this, stool specimens were collected with clean plastic containers. Experienced and trained laboratory technologists assessed the samples to ensure the quality of the investigation. A combination of direct microscopy and the formol-ether concentration technique was used as soon as the specimen collected. The data collection, application of the standard procedure, accuracy of test results was supervised by principal investigators. Some specimens were taken for cross-checking of the accuracy of laboratory results. Filled questionnaires were collected after checking for consistency and completeness.

### Ethics approval and consent to participate

Ethical clearance was obtained from Hawassa University College of Medicine and Health sciences and conducted in accordance with the Declaration of Helsinki and was approved by an institutional review board or ethics committee. Support letter was obtained from the Hawassa city health department. All participants were informed about the purpose, risks, benefit and confidentiality issues related to the study. Participation was on voluntary basis and written informed consent (verbal consent for who cannot read and write respondent) was obtained from each participant. All identified parasites were treated according to the guidelines of the NTD program of Ethiopia ^10^.

### Data analysis

Data entry, cleaning, and analysis were done in SPSS v.23. Descriptive analysis including frequency distribution and the percentage was made to determine the magnitude of IPIs, to describe socio-demographic and clinical characteristics. All factors with a p-value <0.25 in the bivariate logistic regression analysis were a candidate to the multivariable model to control confounding effects. The Hosmer -Lemeshow goodness-of-fit statistic was used to assess whether the necessary assumptions for the application of multiple logistic regression are fulfilled. Odds ratios (OR) with 95% confidence intervals (CI) were calculated. A p-value<0.05 indicated a significant association.

## Results

### Socio-demographic characteristics

A total of 365 pregnant women attending ANC clinic were enrolled in the study. A total of 351 participants were interviewed, yielding a response rate of 96.16%. The age range of the participants was 18–39 years, with the mean (±SD) age of 29.72 (±6.329). Majority of study the participants 313 (89.2%) were married. Regarding educational status, 53 (15.1%) had no formal education whereas 166 (47.3%) had high school education or above. Considering the occupational status of the participants, 140 (39.9%) were unemployed. For the monthly income of the households, 200 (57.0%) earned between 300–1920 ETB, and 151 (43.0%) earned more than 1920 ETB ( Table 1). Individual-level results are available, see *Underlying data* ^13^.

**Table 1.** Socio-demographic characteristics of antenatal care clinic attendants at selected health centers in Hawassa, Southern Ethiopia, 2020.

Category	No.	(%)
Age	
18 – 24 years	83	(23.6)
25 – 34 years	142	(40.5)
35 years or above	126	(35.9)
Marital status		
Married	313	(89.2)
Single	12	(3.4)
Divorced	8	(2.3)
Separated	18	(5.1)
Education status		
No formal education	53	(15.1)
Elementary School	132	(37.6)
High School or Above	166	(47.3)
Occupation		
Non-Employed	140	(39.9)
Merchant	126	(35.9)
Employed	85	(24.2)
Average monthly income		
300–1920 ETB	200	(57.0)
1920 and above	151	(43.0)
Family size		
≤ 4	161	(45.9)
> 4	190	(54.1)

Income based on (HCE, 2016) Exchange rate 1 USD to 29.3673ETB.

### Environmental and behavoural characteristics

A majority of participants (270, 76.9%) were living in a mud-floored house. A total of 188 (53.6%) had a latrine at a household level, and a further 163 (46.4%) shared a latrine with neighbours. The major source of drinking water was pipe water for 339 participants (96.6%). About, 92 (26.2%) still living with domestic animals and pets in the same room. More than half 189 (53.8%) had no handwashing facility to use after using the toilet and 241 (68.7%) were in the habit of eating raw vegetables ( *Extended data*, Table S1) ^13^.

### The prevalence of intestinal parasitic infections

**Figure 1.**
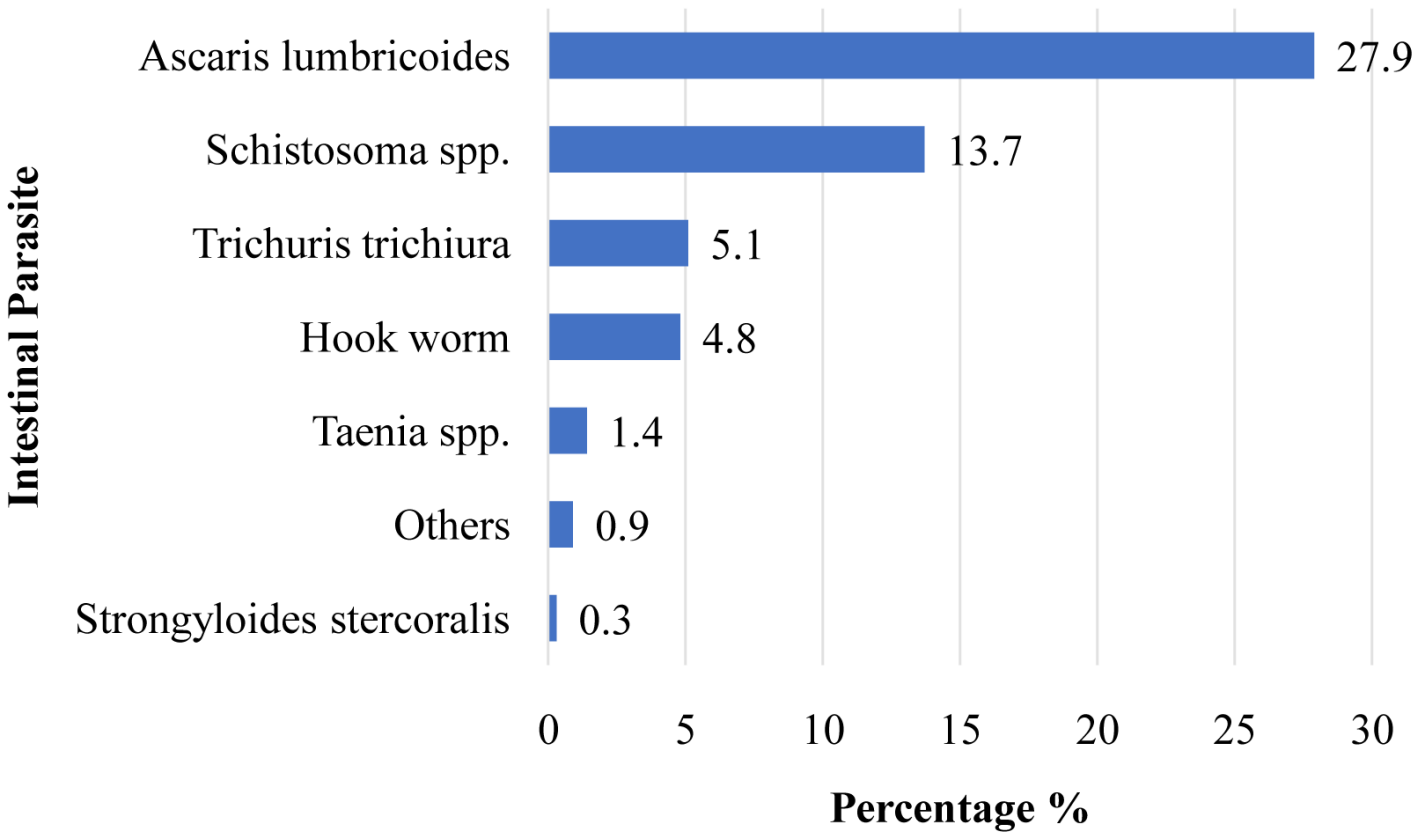
Prevalence of parasitic infections among ANC attendants at health centers in Hawassa, Southern Ethiopia, 2020. (n=351).

The overall number of pregnant women with one or more IPIs was 45.9%, [95% CI: 40.7-51.1] ( *Extended data*, Figure S1) ^13^. The major identified IPs were *A. lumbricoides* (n=98, 27.9%), *Schistosoma* species (n=48, 13.7%), *T. trichiura* (n=18 5.1%), Hookworm (n=17, 4.8%), and *Taenia* species (n=5, 1.4%) ( Figure 1).

### Associated factors for prevalence of intestinal parasitic infections

In multivariate analysis, the education status of respondents, average monthly income, ownership of the latrine, using lake water for washing clothes and habit of eating raw vegetables remained as the determinant of IPIs. The prevalence of IPIs was associated with having no formal education [AOR=2.19, 95% CI: 1.05-4.57] and elementary school-level education [AOR=1.90, 95% CI: 1.11-3.27] as compared with who had high school and above educational status. Having a household monthly income of less than 1920 ETB [AOR=2.06, 95% CI: 1.28-3.31], sharing a latrine with neighbours [AOR=1.83, 95% CI: 1.14-2.93], using lake water for washing clothes [AOR=2.24, 95% CI: 1.34-3.74] and being in the habit of eating raw vegetables [AOR=2.26, 95% CI: 1.30-3.92] were associated with having IPIs when compared to their counterparts ( Table 2).

**Table 2.** Associated factors for parasitic infection among pregnant women at health centers in Hawassa, Southern Ethiopia, 2020.

	Parasitic Infection						
	Yes		No				
	No. (%)		No. (%)		COR (95% CI)	AOR (95% CI)	P-Value
Age							
18 –24 years	32	(38.6)	51	(61.4)	1	1	
25 – 34 years	61	(43.0)	81	(57.0)	1.20(0.69-2.09)	1.04(0.57-1.92)	0.895
35 years or above	68	(54.0)	58	(46.0)	1.87(1.06-3.28)	1.81(0.97-3.38)	0.063
Education status of respondents							
No formal education	32	(60.4)	21	(39.6)	2.99(1.58-5.66)	2.19(1.05-4.57)	0.036 *
Elementary school	73	(55.3)	59	(44.7)	2.43(1.52-3.89)	1.90(1.11-3.27)	0.020 *
High school and above	56	(33.7)	110	(66.3)	1	1	
Occupation of participant							
Unemployed	75	(53.6)	65	(46.4)	2.12(1.21-3.69)	1.75(0.90-3.40)	0.101
Merchant	56	(44.4)	70	(55.6)	1.47(0.83-2.59)	1.28(0.66-2.47)	0.462
Employed	30	(35.3)	55	(64.7)	1	1	
Average monthly income							
< 1920 ETB	107	(53.5)	93	(46.5)	2.07(1.34-3.19)	2.06(1.28-3.31)	0.003 *
1920 and above	54	(35.8)	97	(64.2)	1	1	
Family size							
≤ 4	61	(37.9)	100	(62.1)	1	1	
> 4	100	(52.6)	90	(47.4)	1.82(1.19-2.79)	1.60(0.99-2.59)	0.057
Ownership of the latrine							
Privately owned	71	(37.8)	117	(62.2)	1	1	
Shared with neighbors	90	(55.2)	73	(44.8)	2.03(1.33-3.11)	1.83(1.14-2.93)	0.013 *
Using lake water for washing clothes							
No	103	(54.5)	86	(45.5)	2.15(1.40-3.30)	2.24(1.34-3.74)	0.002 *
Yes	58	(35.8)	104	(64.2)	1	1	
Habit of eating raw vegetables							
No	41	(37.3)	69	(62.7)	1	1	
Yes	120	(49.8)	121	(50.2)	1.67(1.05-2.65)	2.26(1.30-3.92)	0.004 *

*p<0.05. COR: crude odds ratio, AOR: adjusted odds ratio, CI: confidence interval, 1: reference.

## Discussions

This facility-based cross-sectional study revealed that the magnitude of intestinal parasitic infection (IPIs) was 45.9%, [95% CI: 40.7 - 51.1] among pregnant women attending Ante-natal care clinic (ANC). The major identified IPs were *A. lumbricoides* (27.9%), *Schistosoma* spp. (13.7%), *T. trichiura* (5.1%), Hookworm (4.8%) and *Taenia* spp. (1.4%). This study result indicates that 45.9% were found to be infected by one or more IPIs among pregnant women attending ANC. This study result was consistent with the study findings in Nigeria (48.3% infected) ^14^, Bogota, Colombia (41%) ^7^ and in Lalo Kile district, Oromia, Western Ethiopia (43.8%) ^15^. This result was higher when compared to a figure of 13.8% in Kenya ^16^, 12% in another study in Osun state, Nigeria ^17^, 38.7% in Southern Ethiopia ^5^, 21.1% in Northwest Ethiopia ^18^, 31.5% in Northwest Ethiopia ^9^ and 24.7% in the Oromia Region, Ethiopia ^19^. The value in the present study was lower when compared to 70% in a study on the Thai-Burmese border ^8^, 76.2% in Kenya ^20^ and 65% in Gabon ^21^. The difference in findings among various studies could be explained variations in geography, socio-economic condition and cultural practices of study participants.

This study result revealed that the pregnant women who had no formal education were more likely to be exposed to IPIs when compared with those that had high school education and above. This finding was also similar to in Kampala, Uganda ^22^ and northwest Ethiopia ^9^. This may due to a lack of health-related information about prevention, early symptoms and health benefits. In addition to this, those with a household monthly income, less than 1920 ETB was more exposed to IPIs than to their counterparts. This result agrees with another from Western Ethiopia ^15^. This might due to household income directly related to the nutritional status and health status of the individuals.

The prevalence of IPIs was associated with sharing a latrine with neighbours, Habit of eating raw vegetables were more exposed to IPIs when compared to their counterparts. This finding also agreed with that of studies conducted in Kampala, Uganda ^22^, in Northwest Ethiopia ^9^ and in Lalo Kile district, Oromia, Western Ethiopia ^15^. This may due to the sharing latrine with neighbours, no handwashing facility or the absence of proper utilization of latrine and eating uncooked vegetables increases the exposure of the IPIs. This implies that strengthen health education on the proper health education schedule for the ANC attendants and for the community on the proper utilization of the latrine.

This study result shows that using lake water for washing clothes was associated with parasitic infections among pregnant women. A study from Tanzania's Lake Victoria region showed similar results ^23^. Furthermore, Hawassa city was surrounded by lake and most of the low-income pregnant women mainly use for laundry and bathing. It was also used as a source of drinking water in few rural areas. This may lead to greater exposure to schistosomiasis. As such, needs education to change the habits or safe utilization of the lake water.

This study result shows, there was a high prevalence of IPIs; this may lead to causation or aggravation of anaemia via multiple interacting mechanisms ^24^. Hookworms, schistosomiasis and *Trichuris trichiura* lead to intestinal blood loss and reduce appetite and compromise nutrient intake ^15, 25^. Further helminthiasis-inducted intestinal inflammation may limit the absorption of nutrients. This may due to the fact there is an endemic intestinal parasite in the area which exposes the patient to daily activities. The pregnant women had greater roles in the household, which exposes them to waste refuse, gutters and sewage handling. Which aggravated by low income status or economic dependency, low educational status, living condition, and other behavioural related factors and due to the poor level of health-seeking behaviour of the study participants.

This study result indicates that prevention and control activities need to address early detection of IPIs for better prevention, evaluation, and management. In addition to this, health officials and providers needs to collaborate to implement health education and promotion to minimize the impact of the IPIs. This study result also indicates that implementing and strengthening community-based pre-pregnancy care and preparation programs as health extension packages may improve the health of the pregnant women. Government bodies and other stakeholders should work together to improve the household wealth status, create job opportunities for women and maintain affirmative actions for women’s employment. It also needs better strategies to strengthen women's education and employment in the study area. The integration of qualitative research may explore more factors for the future with studies of mixed design at community level.

This study had some potential limitations that might have led to information bias on the part of the respondents on self-reportable risk factors. The study may not establish a causal relationship as we have implemented a cross-sectional study design. 

## Conclusions

This study result shows that nearly half of the pregnant women with IPIs. This indicates that it needs intervention to minimize maternal morbidity and their children. The most common identified IPs were *Ascaris lumbricoides*, *Schistosoma* spp., *Trichuris trichiura*, Hookworm, and *Taenia* spp. The prevalence of IPIs was associated with having no formal education and education to elementary school level as compared with those who had high school and above educational status. Those with a household monthly income less than 1920 ETB, that shared a latrine with neighbours, used lake water for washing clothes and were in the habit of eating raw vegetables were more exposed to IPIs when compared to their counterparts.

## Data availability

### Underlying data

Harvard Dataverse: Replication Data for: Magnitude of Parasitic Infections and Associated Factors among Pregnant Women at Health Facilities in Hawassa, Southern Ethiopia. https://doi.org/10.7910/DVN/III5HA ^13^.

This project contains the following underlying data:

- S2- Additional file 1 Data.csv (raw data associated with this study).

### Extended data

Harvard Dataverse: Replication Data for: Magnitude of Parasitic Infections and Associated Factors among Pregnant Women at Health Facilities in Hawassa, Southern Ethiopia. https://doi.org/10.7910/DVN/III5HA ^13^.

This project contains the following extended data:

- S1-Additional file Table.doc (Table S1).
- S3 Additional file English qoestionner.doc (questionnaire used in this study; English version).
- S4- Additional file Figure 1.docx (Figure S1).
- The prevalence of intestinal parasitic output on Sheet 2 and Multivariable logistic regration output on Sheet 1.tab (descriptive statistics derived from raw data).

### Reporting guidelines

Harvard Dataverse: STROBE checklist for ‘Magnitude of parasitic infections and associated factors among pregnant women at health facilities in Hawassa, Southern Ethiopia’. https://doi.org/10.7910/DVN/III5HA ^13^.

Data are available under the terms of the Creative Commons Zero "No rights reserved" data waiver (CC0 1.0 Public domain dedication). 
