# Supplementary material for: Magnitude of parasitic infections and associated factors among pregnant women at health facilities in Hawassa, Southern Ethiopia
Source: F1000Res. 2021 Feb 17;10:122. [Version 1] doi: 10.12688/f1000research.27584.1 (PMC8182691; doi:10.12688/f1000research.27584.1)
Supplement: Peer review history

# Peer review: Reviewer response for version 1

**DOI:** 10.5256/f1000research.30485.r86426

**Published:** 2021-06-04

**Authors:** Abdelhakam G. Tamomh

**Affiliations:** 1 Department of Parasitology and Medical Entomology, Faculty of Medical Laboratory Sciences, University of El Imam El Mahdi, Kosti, Sudan

The manuscript about IPIs and associated risk factors among pregnant women is interesting. However, I have the following comments:

 The **title** is well defined and a little change should be considered as, ‘’Prevalence rate of intestinal parasitic infections and associated risk factors among pregnant women at health facilities in Hawassa, Southern Ethiopia’’.

 The **abstract** should be revised and especially the conclusion should be more concentrated.

 The **introduction** should include the burden of intestinal parasitic infections among pregnant women globally.

 Pregnant women are more susceptible to parasitic infections that attributed to the immunological changes during pregnancy, therefore the intestinal coccidian parasites were missing in this study and should be mentioned in the limitation of the study section.

 In **Material and Methods**, direct microscopy - please change it to direct wet preparation.

 Besides a combination of direct wet preparation and formol-ether concentration techniques, a staining technique was missed, such as the modified ZN staining technique which is used only to detect intestinal *Coccidia* ( *Cryptosporidium* species, *Isospora belli* and *Cyclospora cayetanensis*), please mention it in the limitations of the study section?

 The gestation age of the pregnant women (either in the first, second, or third trimester) was not explained.

 Additionally, the gravidity of pregnant women not addressed (such as the multigravid women may have more risk than primigravid women).

 The **results** support the prevalence and risk factors, therefore, the term prevalence rate is better than Magnitude.

 If the information related to the age of gestation and the gravidity is addressed please added it to table 2.

 In **the discussion** section, the authors discussed the results well.

 In **conclusion**, after mentioning the importance of some results, please conclude the outcome of the paper.

 I think after making these corrections the paper will be approved. I have attempted to add some corrections to this manuscript. Hopefully, this will be helpful for the authors.

Is the work clearly and accurately presented and does it cite the current literature?

Yes

If applicable, is the statistical analysis and its interpretation appropriate?

Yes

Are all the source data underlying the results available to ensure full reproducibility?

Yes

Is the study design appropriate and is the work technically sound?

Yes

Are the conclusions drawn adequately supported by the results?

Yes

Are sufficient details of methods and analysis provided to allow replication by others?

Yes

Reviewer Expertise:

Medical Parasitology, Infectious diseases, Tropical diseases, Clinical laboratory diagnostics, Clinical Parasitology, Clinical immunology.

I confirm that I have read this submission and believe that I have an appropriate level of expertise to confirm that it is of an acceptable scientific standard.

# Peer review: Reviewer response for version 1

**DOI:** 10.5256/f1000research.30485.r79715

**Published:** 2021-04-12

**Authors:** Guéladio Cissé

**Affiliations:** 1 Swiss Tropical and Public Health Institute (Swiss TPH), Basel, Switzerland; 2 University of Basel, Basel, Switzerland

**Title:**

 Why the use of "Magnitude"? The choice could be explained in the text (Introduction), if the prevalence as the main outcome presented in Figure 1 and Table 2 was not appropriate in the title. 

**Introduction:**

 The problem statement is not made in a strong logical order. The text should prepare the reader to the interest of doing this study in a region and from health facilities.

 Good to check the English: some sentences are not complete (no verb, etc...). In particular, paragraph 3 needs to be re-ordered. The first sentence does not have a verb and it seems not make sense here for the problem statement. It would be more logical to start with the second part of the second sentence. (Note: starting with a capital letter "There is ..." is misleading, making difficult to understand the first part).

 It would be better to start first by setting the national level burden and then the gap of knowledge or the diversity of current results at the regional levels; this would show the interest of this regional based study. It is not only important to identify the magnitude of IPIs but to know better the situation in regions.

**Methods:**

 Need more information on the study site /region (e.g. climate, spatial location, poverty indicators, education status, parasites sensitive environment and ecosystems in the area, etc...) - Why the study is done/relevant in this region?

**Results:**

 2 tables and 3 figures; main results are about "prevalence" and risk factors.

**Discussion:**

 Sometimes it could be possible to reduce the extended repetition of some results. Also, when a reference is mentioned from other studies (stating similar results or differences), it would be good to provided the exact results from the other study(ies). To avoid saying that the results are similar or different from a study and giving only the reference.

Is the work clearly and accurately presented and does it cite the current literature?

Yes

If applicable, is the statistical analysis and its interpretation appropriate?

Yes

Are all the source data underlying the results available to ensure full reproducibility?

Yes

Is the study design appropriate and is the work technically sound?

Yes

Are the conclusions drawn adequately supported by the results?

Yes

Are sufficient details of methods and analysis provided to allow replication by others?

Yes

Reviewer Expertise:

Intestinal parasitic infections related research, water quality and health, climate change and health, WASH and nutrition integrated approaches

I confirm that I have read this submission and believe that I have an appropriate level of expertise to confirm that it is of an acceptable scientific standard.
